# Pre-announcement of symbiotic guests: transcriptional reprogramming by mycorrhizal lipochitooligosaccharides shows a strict co-dependency on the GRAS transcription factors NSP1 and RAM1

**DOI:** 10.1186/s12864-015-2224-7

**Published:** 2015-11-23

**Authors:** Natalija Hohnjec, Lisa F. Czaja-Hasse, Claudia Hogekamp, Helge Küster

**Affiliations:** Institut für Pflanzengenetik, Abt. IV - Pflanzengenomforschung, Leibniz Universität Hannover, Herrenhäuser Str. 2, D-30419 Hannover, Germany; Present address: Max Planck Genome Centre Cologne, Carl-von-Linné-Weg 10, D-50829 Köln, Germany

**Keywords:** Arbuscular mycorrhiza, GRAS transcription factor, Lipochitooligosaccharide, *Medicago* GeneChip, NSP1, Pre-symbiotic signaling, RAM1, Transcriptional reprogramming

## Abstract

**Background:**

More than 80 % of all terrestrial plant species establish an arbuscular mycorrhiza (AM) symbiosis with *Glomeromycota* fungi. This plant-microbe interaction primarily improves phosphate uptake, but also supports nitrogen, mineral, and water aquisition. During the pre-contact stage, the AM symbiosis is controled by an exchange of diffusible factors from either partner. Amongst others, fungal signals were identified as a mix of sulfated and non-sulfated lipochitooligosaccharides (LCOs), being structurally related to rhizobial nodulation (Nod)-factor LCOs that in legumes induce the formation of nitrogen-fixing root nodules. LCO signals are transduced via a common symbiotic signaling pathway (CSSP) that activates a group of GRAS transcription factors (TFs). Using complex gene expression fingerprints as molecular phenotypes, this study primarily intended to shed light on the importance of the GRAS TFs NSP1 and RAM1 for LCO-activated gene expression during pre-symbiotic signaling.

**Results:**

We investigated the genome-wide transcriptional responses in 5 days old primary roots of the *Medicago truncatula* wild type and four symbiotic mutants to a 6 h challenge with LCO signals supplied at 10^-7/-8^ M. We were able to show that during the pre-symbiotic stage, sulfated Myc-, non-sulfated Myc-, and Nod-LCO-activated gene expression almost exclusively depends on the LysM receptor kinase NFP and is largely controled by the CSSP, although responses independent of this pathway exist. Our results show that downstream of the CSSP, gene expression activation by Myc-LCOs supplied at 10^-7/-8^ M strictly required both the GRAS transcription factors RAM1 and NSP1, whereas those genes either co- or specifically activated by Nod-LCOs displayed a preferential NSP1-dependency. RAM1, a central regulator of root colonization by AM fungi, controled genes activated by non-sulfated Myc-LCOs during the pre-symbiotic stage that are also up-regulated in areas with early physical contact, e.g. hyphopodia and infecting hyphae; linking responses to externally applied LCOs with early root colonization.

**Conclusions:**

Since both RAM1 and NSP1 were essential for the pre-symbiotic transcriptional reprogramming by Myc-LCOs, we propose that downstream of the CSSP, these GRAS transcription factors act synergistically in the transduction of those diffusible signals that pre-announce the presence of symbiotic fungi.

**Electronic supplementary material:**

The online version of this article (doi:10.1186/s12864-015-2224-7) contains supplementary material, which is available to authorized users.

## Background

More than 80 % of all land plant species enter an arbuscular mycorrhiza (AM) symbiosis with *Glomeromycota* fungi. This plant-microbe interaction primarily improves phosphate uptake, but also contributes to the acquisition of nitrogen, minerals, and water [1].

AM host plants secrete strigolactones that stimulate fungal metabolism and promote pre-symbiotic hyphal branching [2, 3]. In return, AM fungal recognition by the plant is thought to be mediated by Myc-factors that signal the presence of symbiotic microbes. The chemical structure of one class of diffusible factors from AM fungi was revealed by Maillet et al. [4], who identified a mixture of sulfated (s) and non-sulfated (ns) lipochitooligosaccharide (LCO) molecules in fungal exudates. Although the N-linked acyl chain is different [4], Myc-LCOs are structurally related to rhizobial nodulation (Nod)-factor LCOs. After perception by rhizodermal LysM receptor kinase heterodimers (NFP/LYK3 in *Medicago truncatula*), these LCOs mediate bacterial infection and the induction of nodule primordia [5]. The general similarity between rhizobial and AM fungal LCOs indiates that rhizobia adopted a pre-existing, much older mycorrhizal signaling program to initiate host responses [6].

In addition to being involved in mycorrhization and nodulation, microbial LCOs stimulate the formation of lateral roots [4]. Since the activation of this basic developmental process [4] and the transcriptional responses of roots towards Myc-LCOs require NFP [7], recognition of AM fungal LCO signals is probably mediated by this LysM receptor. However, since NFP-type receptors are missing a functional kinase domain [1], another kinase is additionally required for signal transduction. One such candidate was proposed to be LYK3, the second component of the heterodimeric Nod-LCO receptor and a *M. truncatula* orthologue of the rice chitin receptor OsCERK1 [8].

Perception of Myc-LCO signals leads to characteristic Ca^2+^-oscillations in rhizodermal cells [9, 10]. In *M. truncatula*, this Ca^2+^ spiking depends on the LRR (leucine-rich repeat) receptor kinase DMI2 (doesn't make infections 2) and on DMI1, a potassium channel of the nuclear membrane. Ultimately, the Ca^2+^/calmodulin-dependent protein kinase DMI3, together with its interacting transcription factor Cyclops, receives the spiking pattern [11, 12]. Since mycorrhizal as well as rhizobial infection is controled by the same set of DMI proteins, this signal cascade is referred to as the common symbiotic signaling pathway (CSSP).

Apart from Myc-LCOs, short chain chitooligosaccharides (COs) were identified as AM fungal signals that induce Ca^2+^ spiking [9, 10]. In root organ cultures [9], the level of CO4 and CO5 was enhanced by plant-derived strigolactones, and these CO versions induced a spiking pattern similar to exudates from germinating spores, being more persistent than that observed for Myc-LCOs. This response required DMI1 and DMI2, but, in contrast to Myc-LCOs, was NFP-independent, suggesting a role for other LysM kinases [9]. Similar to Myc-LCO perception, homologues of the CERK1 chitin receptor might be involved here [13].

In addition to diffusible Myc-factors acting at a distance [10, 14–17], several lines of evidence point to signals that require physical contact between fungal hyphae and host roots [18–20]. Such contact signals coordinate a specific re-differentiation of rhizodermal and cortical cells, once hyphopodia attach to the root surface. The most remarkable cytological response of the infected host cells is the development of a cytoplasmatic pre-penetration apparatus (PPA). Upon hyphopodium formation, this structure is established by each successively infected cell and guides fungal hyphae towards the inner cortex [21]. Here, they proliferate in the apoplast and ultimately form intracellular, highly branched arbuscules that serve as the major interface for nutrient exchanges [22].

Global transcriptional changes in *M. truncatula* AM roots were studied extensively, resulting in a comprehensive overview of symbiotic gene expression on the tissue and cellular level [23–31]. In contrast to these studies that investigated colonized roots, genome-wide analyses of pre-symbiotic gene expression responses to diffusible Myc-signals are still limited [32]. Based on *Medicago* GeneChip hybridizations, we previously reported on the LCO-related transcriptional changes in 5 days old primary roots of the *M. truncatula* wild type [7], challenging roots for 6 or 24 h (h) with 10^−7^ (nsMyc-) or 10^-8^ M (sMyc-, Nod-) LCOs. A sufficient activation of symbiotic signaling at these concentrations was validated by the histological detection of epidermal *MtEnod11* [33] expression. The complex transcriptional responses towards Myc-LCOs were almost absolutely dependent on NFP and largely on DMI3, indicating that during the pre-symbiotic stage, AM fungal LCO signals are predominantly transduced via the CSSP [7]. In a comparable experimental setup, Camps et al. [34] recently studied the response of 4 days old primary *M. truncatula* roots in the wild type and in *dmi3-1* mutants. The most notable differences between the two studies are the shorter incubation time of 2–4 h and the usage of 10–100 fold higher (10^−6^ M) concentrations of Myc-LCOs by Camps et al. [34], together leading to an identification of more differentially regulated genes. In additon, RNAseq was applied, which allowed to measure the expression of genes not represented on *Medicago* GeneChips. Similar to 10^-7/-8^ M Myc-LCO concentrations [7], Camps et al. [34] demonstrated that LCO-signaling is predominantly mediated by DMI3 also at 10^−6^ M, although a DMI3-independent signaling pathway was defined as well.

In addition to studying CSSP-dependency, Camps et al. [34] also looked into Myc-LCO activated gene expression in *nsp1-1* mutants. Interestingly, a high proportion of the Myc-LCO induced, DMI3-dependent genes appeared to require the GRAS transcription factor (TF) NSP1, as already reported for a limited number of genes [7, 35]. NSP1 acts immediately downstream of the CSSP and was originally thought to be exclusively involved in nodulation [36–38]. Delaux et al. [35] and Takeda et al. [39] had nevertheless reported that NSP1-deficient legume mutants displayed a reduced AM fungal colonization. A comparable, non-essential function for mycorrhization was also demonstrated for the NSP1-interactor NSP2 [4], another GRAS TF initially thought to be exclusively required for nodulation [6, 40]. It has to be noted that both NSP1 and NSP2 play a role in strigolactone biosynthesis [41], providing further evidence for a link between NSP GRAS TFs and AM formation.

Recently, the GRAS TF RAM1 was identified in a screen for AM-deficient mutants [42, 43]. Similar to NSP1, RAM1 interacts with NSP2 [42, 44], illustrating that nodulation and mycorrhization are controled by overlapping sets of regulators [10]. Since in contrast to NSP1, RAM1 was not described to be required for the transduction of Nod-signals [43, 45, 46], RAM1 is currently regarded both as an essential and a specific component of Myc-signaling. In later stages of AM, RAM1 plays a role in the development of functional arbuscules both in *M. truncatula* and petunia [43, 45, 46], a function consistent with the *MtRAM1* promoter activity in cortical cells [47].

While putative receptors (NFP, LYK3) and components of the CSSP mediating the pre-symbiotic transduction of LCO signals have clearly been identified [4, 7–10, 34], the interdependency of the key regulators that activate LCO-related transcriptional networks immediately downstream of the CSSP remains to be adressed. To shed light on the relative importance of the GRAS TFs NSP1 and RAM1 for LCO-activated gene expression particularly during the pre-symbiotic stage, we obtained global transcription patterns of 5 days old *nsp1-1* and *ram1-1* plantlet roots after a 6 h challenge with Myc- and Nod-LCOs. As in a previous study [7], we used 10^-7/-8^ M LCO concentrations, in order to be as close as possible to the lower concentrations of Myc-LCOs that likely exist in soils and to avoid over-activation of gene expression by higher signal concentrations. Together with the transcriptional responses of *nfp-1* and *dmi3* mutants, the expression fingerprints obtained served as molecular phenotypes to specify the requirement for nodulation- and mycorrhization-related GRAS TFs during the initial transduction of LCO signals, prior to a contact between the symbionts. Our findings provide evidence that in contrast to Nod-LCO signaling that predominantly relies on NSP1, the presence of both NSP1 and RAM1 is essential for almost all Myc-LCO related gene expression activation in the pre-symbiotic stage. Moreover, our results link the pre-symbiotic responses with the transcriptional reprogramming during AM fungal contact and early root colonization, a process largely blocked in *ram1-1* mutants. We propose a model, where downstream of the CSSP, the RAM1 and NSP1 TFs act synergistically in the transduction of diffusible Myc-LCO signals.

## Results

We here present an integrated view of the global transcriptional responses in primary roots of the *M. truncatula* wild type A17 as well as the *nfp-1*, *dmi3*, *nsp1-1*, and *ram1-1* mutants towards AM fungal (sMyc-, nsMyc-, s/nsMyc-) and rhizobial (Nod-) LCOs, using concentrations of 10^−7^ (nsMyc-) or 10^−8^ M (sMyc-, Nod-LCOs), respectively. For all LCO treatments, we used 5 days old seedlings, being in a stage of development where the first trifoliate was just initiated. Using *nsp1-1* and *ram1-1* mutants that lack functional NSP1 and RAM1 GRAS TFs, our experiments build on a study of Myc-LCO induced gene expression in the wild type as well as *nfp-1* and *dmi3* mutants [7]. Acting downstream of the CSSP, the NSP1 and RAM1 regulators can be considered as key jigsaw pieces of early Myc-LCO signaling. To identify Myc-LCO specific effects, the transcriptional responses towards Nod-LCOs in the wild type [7] were now complemented with results for *nfp-1*, *dmi3*, *nsp1-1*, and *ram1-1* mutants as well. In our experimental conditions that rely on the terminal 2 to 2.5 cm of 5 days old primary roots excluding the root meristem, these concentrations led to an easily visible epidermal induction of the *MtEnod11* promoter by all LCOs tested, including the least efficient nsMycLCOs [7]. Together with the marked DMI3-dependency of most gene expression responses to 10^-7/-8^ M Myc-LCOs [7], this indicates that symbiotic Ca^2+^ spiking was activated to a sufficient extent in our conditions. Due to the fact that gene expression responses towards all Myc-LCOs occurred much stronger after 6 h as compared to 24 h [7], we here focus on this early time point. A compilation of all gene expression data is available as Additional file 1 (Table S1) , and will be discussed in detail in the subsequent chapters.

### Activation of symbiotic marker genes by Myc- and Nod-LCOs displays a differential requirement for the GRAS transcription factors NSP1 and RAM1

To deepen our understanding of the transcriptional activation by symbiotic LCOs, we first had a look at the expression properties of 26 genes related to symbiotic signaling (Additional file 2: Table S2). This analysis revealed that *MtNFP*, *MtLYK3*, and all components of the CSSP (*MtDMI1*, *MtDMI2*, *MtDMI3*) as well as *MtCyclops* were not activated in the wild type by any symbiotic LCO tested.

**Fig. 1:**
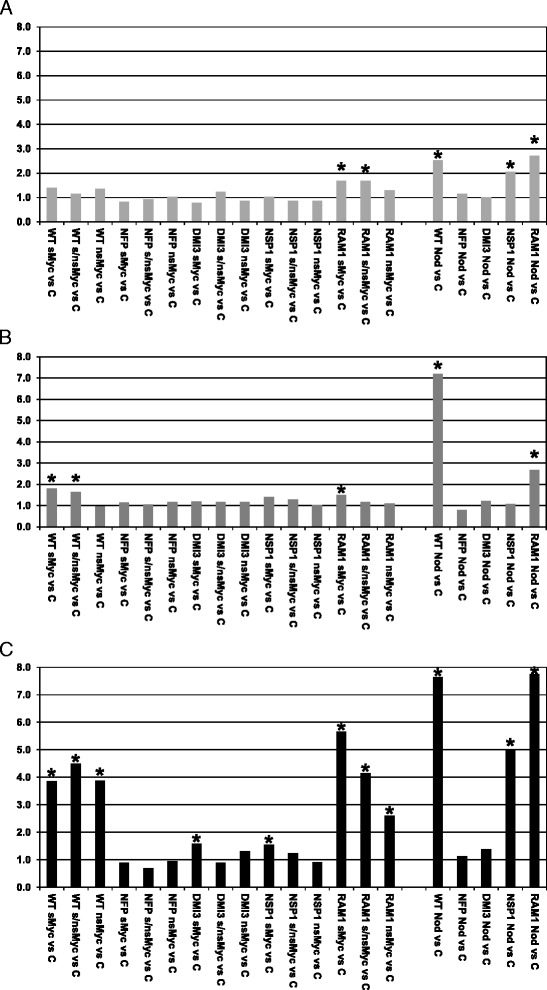
Expression of symbiotic marker genes in response to Myc- and Nod-LCOs. Expression of *MtNsp1* (**a**), *MtEnod11* (**b**), and *MtVapyrin* (**c**) in *M. truncatula* wild type A17 (WT), *nfp-1* (NFP), *dmi3* (DMI3), *nsp1-1* (NSP1)*,* and *ram1-1* (RAM1) plantlet roots challenged for 6 h with 10^−8^ M sMyc-LCOs (sMyc), 10^−7^ M nsMyc-LCOs (nsMyc), a mixture of 10^−8^ M sMyc- and 10^−7^ M nsMyc-LCOs (s/nsMyc), and 10^−8^ M Nod-LCOs (Nod). Ratios of up-regulation in comparison to non-treated controls (**c**) is shown. An at least 1.5-fold (*p* < 0.05) up-regulation is denoted by asterisks

Whereas *MtNSP2* was slightly activated only after a 24 h treatment with Nod-LCOs, *MtNSP1* was 2.5-fold up-regulated in the wild type by this factor already after 6 h (Fig. 1). This up-regulation depends on NFP and DMI3, but was independent of both NSP1 itself (2.1-fold activation) and RAM1 (2.7-fold activation). *MtNSP1* in addition displayed a tendency for activation by Myc-LCOs (1.2 to 1.4-fold, Fig. 1), which was absent in *nsp1-1*, but still detectable in *ram1-1* mutants (1.3 to 1.7-fold, Fig. 1). The induction of *MtNSP1* by Myc-LCOs thus appears to be independent of RAM1, an observation in line with results Delaux et al. [35] obtained for a 10^−8^ M s/nsMyc-LCO mixture.

A well-characterized marker gene responding to LCOs is *MtEnod11* [4, 33]. This gene was found activated most strongly by Nod- (7.2-fold) and only slightly by sMyc- and s/ns-MycLCOs (1.8-fold and 1.6-fold, respectively) in wild type plantlet roots ([7], Fig. 1). Whereas *MtEnod11*-activation by Nod-LCOs was not detected in *nfp-1*, *dmi3*, and *nsp1-1* mutants, this gene was still up-regulated in the *ram1-1* line, although somewhat lower (2.7-fold, Fig. 1). Activation of *MtEnod11* by sMyc-LCOs follows a similar tendency (1.5-fold activation by sMyc-LCOs only in *ram1-1* mutants, Fig. 1). The RAM1 independency of *MtEnod11* expression in response to sMyc- and Nod-LCOs conforms to the histological results of Sun et al. [10]. As reported previously [7, 35], no *MtEnod11* induction by nsMyc-LCOs was detected in the pooled root samples used here, probably due to a quenching of the epidermal expression observed by reporter gene studies [7].

A prominent example for a symbiosis-specific gene relevant both for AM fungal and rhizobial infection is *MtVapyrin*, an ankyrin repeat protein involved in membrane trafficking [31, 47, 48]. In line with a report by Sun et al. [10], who used different concentrations of LCOs applied to only 2 days old seedlings, this gene was also found to be up-regulated between 3.9- and 7.7-fold by all symbiotic LCOs in the wild type in our conditions ([7], Fig. 1). Similar to many other LCO-induced genes [7], *MtVapyrin* activation by fungal LCOs strictly depends on NFP (Fig. 1). Whereas DMI3 and NSP1 were similarly required for an expression activation by nsMyc- and s/nsMyc-LCOs, sMyc-LCOs still induced *MtVapyrin* in *dmi3* and *nsp1-1* mutant roots, although at a strongly reduced level (1.6- and 1.5-fold; Fig. 1). In contrast, *MtVapyrin* up-regulation was independent of RAM1 for all fungal LCOs tested, reminiscent of the detection of *Vapyrin* expression in mycorrhizal roots of petunia *ram1-1* mutants [47]. With respect to Nod-LCOs, *MtVapyrin* induction also required NFP and DMI3, but was independent of both NSP1 and RAM1 (Fig. 1). The differential *MtVapyrin* expression observed in wild type, *nfp-1*, *dmi3*, and *nsp1-1* roots was comparable to results obtained for 10^-10^ M Nod-LCOs [48]. It thus appears that while the up-regulation of *MtVapyrin* is largely NSP1-dependent in response to Myc-, it is NSP1-independent in response to Nod-LCOs.

Together, the activation of symbiotic marker genes by Myc- and Nod-LCOs points to a differential requirement for the symbiotic GRAS TFs NSP1 and RAM1, indicating that these regulators might be of different relative importance for Myc- and Nod-LCO signaling in general.

### Gene expression activation by Myc-LCOs strongly depends on the two GRAS transcription factors NSP1 and RAM1

We previously reported on the transcriptional responses of *M. truncatula* wild type A17, *nfp-1*, and *dmi3* plantlet roots towards Myc-LCOs, leading to the conclusion that the LysM receptor kinase NFP is almost absolutely essential for the perception of Myc-LCO signals and that the majority of Myc-LCO specific gene expression is activated via the CSSP [7].

**Fig. 2:**
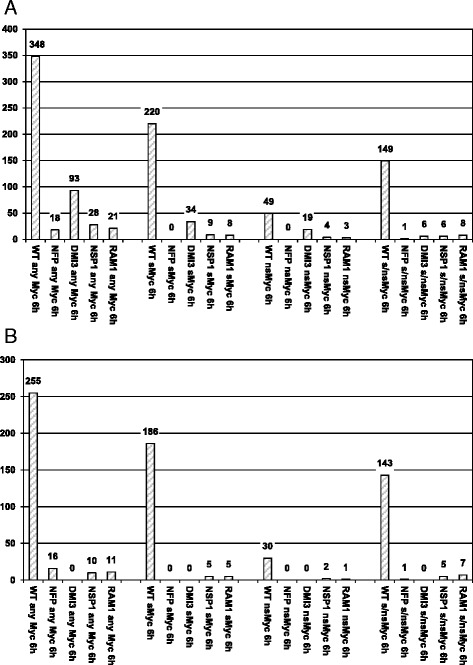
Myc-LCO related gene expression in the *M. truncatula* wild type and in four symbiotic mutants. **a** Number of genes at least 1.5-fold (*p* < 0.05) up-regulated in *M. truncatula* wild type A17 (WT), *nfp-1* (NFP), *dmi3* (DMI3), *nsp1-1* (NSP1)*,* and *ram1-1* (RAM1) plantlet roots challenged for 6 h with 10^−8^ M sMyc-LCOs (sMyc), 10^−7^ M nsMyc-LCOs (nsMyc), or a mixture of 10^−8^ M sMyc- and 10^−7^ M nsMyc-LCOs (s/nsMyc). None of these genes is activated by Nod-LCOs at this cutoff (Additional file 3: Table S3). On the left, the three Myc-LCO specific data columns are summed up, regardless of individual Myc-LCOs. Values do not add up due to an overlapping activation of transcription by different LCOs. The number of genes induced by Myc-LCOs in the wild type still activated in four symbiotic mutants are shown to the right of the wild type columns. Expression of Myc-LCO related genes in *nfp-1* and *dmi3* plantlet roots reported by [7] were included to facilitate comparisons. **b** Genes still activated in *dmi3* mutants were subtracted (Additional file 4: Table S4) to highlight CSSP-dependent effects

In order to investigate processes occuring downstream of the CSSP, we studied the transcriptional responses towards sMyc-, nsMyc-, and s/nsMyc-LCOs in *nsp1-1* and *ram1-1* plantlet roots. To allow a direct comparison, the resulting gene expression patterns were analyzed together with previous data for wild type, *nfp-1*, and *dmi3* roots [7]. These analyses revealed that out of 348 genes activated at least 1.5-fold (*p* < 0.05) by Myc-LCOs and not by Nod-LCOs after 6 h in the wild type ([7], referred to as Myc-LCO related genes here, Additional file 3: Table S3), only 21 were still up-regulated at these cutoffs by any Myc-LCO or Myc-LCO combination in *ram1-1*, equivalent to an appr. 94 % reduction (Fig. 2). This observation fits well to the strongly impaired AM fungal root colonization observed in this mutant [42, 45]. Strikingly, a comparable reduction of gene expression responses (appr. 92 %) was also observed in the *nsp1-1* mutant, where only 28 genes remained activated by any Myc-LCO or Myc-LCO combination (Fig. 2). Although NSP1 was recently described to be relevant for mycorrhization [34–36], the extent of reduction of gene expression responses towards Myc-LCOs was unexpected.

**Fig. 3:**
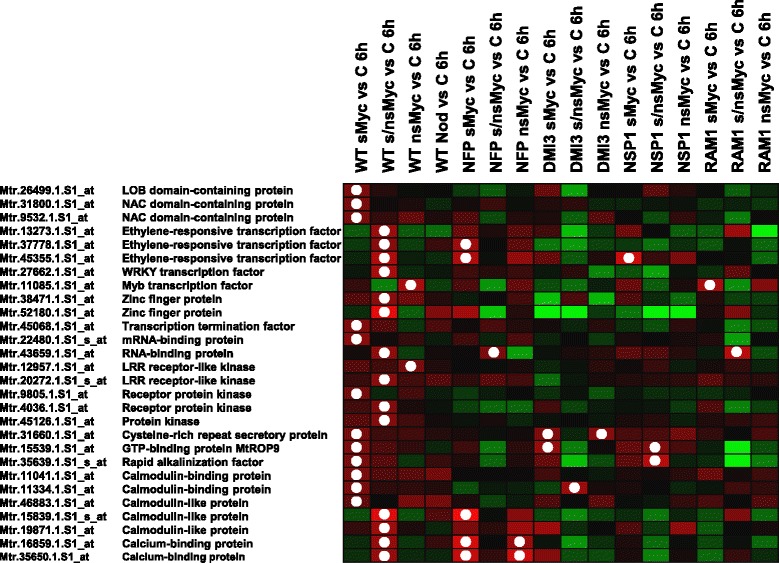
Expression of Myc-LCO related genes encoding signaling components in the *M. truncatula* wild type and in four symbiotic mutants. Subset of 28 genes encoding transcription factors or signaling-related proteins and being rapidly activated in *M. truncatula* wild-type roots after a 6 h treatment with 10^−8^ M sMyc-LCOs (sMyc), 10^−7^ M nsMyc-LCOs (nsMyc), or a mixture of both (s/nsMyc). None of these genes is significantly activated by 10^−8^ M Nod-LCOs (Nod) after 6 h [7]. Expression responses after 6 h in the wild type (WT) as well as the *nfp-1* (NFP), *dmi3* (DMI3), *nsp1-1* (NSP1), and *ram1-1* (RAM1) mutants is visualized as a heat map, with shades of red representing up- and shades of green down-regulation. An at least 1.5-fold (*p* < 0.05) up-regulation is indicated by a white dot. Expression data were scaled to the maximum of a 2.5-fold regulation using the Genesis software [61]

The concomitant importance of NSP1 and RAM1 for signaling downstream of the CSSP prompted us to focus on 31 Myc-LCO related genes encoding transcriptional regulators or components of signal transduction reported previously [7]. After removing duplicate probes identified from the most recent *M. truncatula* genome annotation (Additional file 1: Table S1), transcription of the remaining 28 genes in wild type and mutant roots was visualized in Fig. 3. In addition to a strong reduction of expression in *nfp-1* and *dmi3* mutant roots, the 28 Myc-LCO related genes in general were not significantly up-regulated in the *nsp1-1* and *ram1-1* background. In all but two cases, a significant activation in mutant roots only occurred in response to another LCO or LCO combination as in the wild type, probably indicating some level of deregulation in the symbiotic mutants (Fig. 3). The general absence of an expression activation comparable to the wild type indicates that the signaling-related genes mentioned above encode components of Myc-LCO signaling activated not only via the CSSP, but subsequently via the GRAS transcription factors NSP1 and RAM1.

In total, only 4 Myc-LCO related genes were still activated both in *nsp1-1* and *ram1-1* mutants. Whereas activation of all four genes required NFP, three were DMI3-independent (Additional file 2: Table S2), indicating that some Myc-LCO related genes can be activated independent of the CSSP by an alternative pathway that also doesn't require NSP1 and RAM1.

To exclude a bias from CSSP-independent effects, we subtracted all genes still activated in *dmi3* mutants, arriving at 255 out of 348 Myc-LCO related genes whose expression relies on the CSSP (Additional file 4: Table S4). Apart from a requirement for NFP (94 % reduction, Fig. 2), the strong dependency on both NSP1 and RAM1 was again evident, with gene expression activation by Myc-LCOs being reduced by 96 % in each case (Fig. 2). This strong NSP1-/RAM1-codependency is also evident, if the effects are monitored separately for sMyc-, nsMyc-, or s/nsMyc-LCO induced genes (Fig. 2).

Interestingly, the co-dependency on NSP1 and RAM1 observed for genes up-regulated by Myc-LCOs is also a characteristic of genes down-regulated by a treatment with these symbiotic signals (Additional file 1: Table S1). In conclusion, our results demonstrate that the two symbiotic GRAS TFs NSP1 and RAM1 are both essential for almost all gene expression responses towards a 6 h application of 10^-7/-8^ M Myc-LCOs.

### Genes co-activated by Myc- and Nod-LCOs display a preferential requirement for the GRAS transcription factor NSP1

**Fig. 4:**
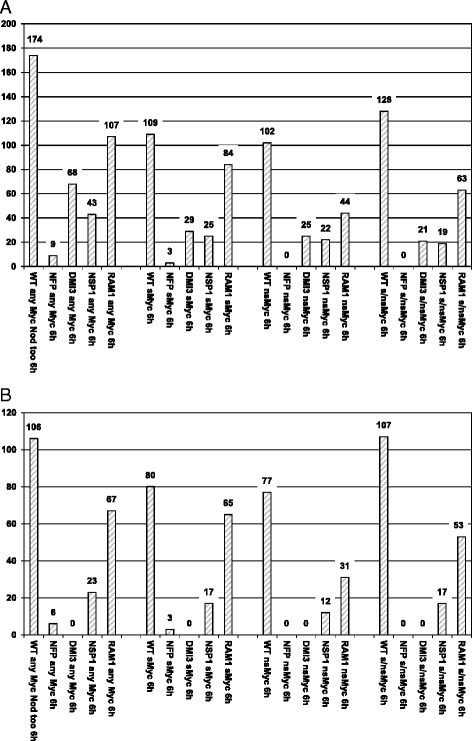
Sym-LCO related gene expression in the *M. truncatula* wild type and in four symbiotic mutants. **a** Number of genes at least 1.5-fold (*p* < 0.05) up-regulated in *M. truncatula* wild type A17 (WT), *nfp-1* (NFP), *dmi3* (DMI3), *nsp1-1* (NSP1)*,* and *ram1-1* (RAM1) plantlet roots challenged for 6 h with 10^−8^ M sMyc-LCOs (sMyc), 10^−7^ M nsMyc-LCOs (nsMyc), or a mixture of 10^−8^ M sMyc- and 10^−7^ M nsMyc-LCOs (s/nsMyc). These genes were also activated by Nod-LCOs at this cutoff (Additional file 3: Table S3). On the left, the three Myc-LCO specific data columns are summed up, regardless of individual Myc-LCOs. Values do not add up due to an overlapping activation of transcription by different LCOs. The number of genes induced by Myc-LCOs in the wild type still activated in four symbiotic mutants are shown to the right of the wild type columns. **b** Genes still activated in *dmi3* mutants were subtracted (Additional file 4: Table S4) to highlight CSSP-dependent effects

To further investigate the differential NSP1- and RAM1-dependency of Myc-LCO-related transcription, we studied the expression in *nfp-1*, *dmi3*, *nsp1-1*, and *ram1-1* mutants of those 174 genes activated at least 1.5-fold (*p* < 0.05) not only by Myc-, but also by Nod-LCOs after 6 h in the wild type ([7], referred to as Sym-LCO related genes here, Additional file 3: Table S3). This analysis revealed that gene expression responses (using the cutoffs mentioned above) were reduced to comparable levels in *nfp-1* and *dmi3*-mutants (appr. 95 % and 61 %, respectively, Fig. 4) as observed for Myc-LCO related genes (95 % and 73 %, respectively, Fig. 2), confirming that these genes are primarily activated via the CSSP. Interestingly, the *nsp1-1* and *ram1-1* mutants now displayed a lower and clearly differential level of reduction (75 % and 39 %, respectively, Fig. 4; compared to 92 % and 94 % for Myc-LCO related genes, respectively, Fig. 2), allowing the conclusion that genes co-activated by Nod-LCOs (Fig. 4) show a higher dependency on NSP1 as compared to RAM1. This was most apparent for those genes activated by sMyc-LCOs, sharing the highest structural similarity to Nod-LCOs. Here, from 109 genes co-activated by sMyc- and Nod-LCOs, 84 remained induced by sMyc-LCOs in *ram1-1* mutants, while only 25 displayed activation by these LCOs in the *nsp1-1* background. In contrast, the difference in reduction of responses to nsMyc-LCOs was less pronounced in the *nsp1-1* and *ram1-1* mutants (Fig. 4). Similar to the situation for Myc-LCO related genes, exclusion of those Sym-related genes activated in *dmi3* mutants led to comparable results (Additional file 4: Table S4, Fig. 4).

Based on these results we infer that transcriptional activation is less RAM1-dependent, if it is triggered not only by Myc-, but also by Nod-LCOs. This observation is corroborated by an analysis of the 106 genes identified by Camps et al. [34] being commonly activated by Myc- and/or Nod-LCOs both at 10^-7/-8^ [7] and 10^−6^ M [34]. From the 67 genes of this set co-induced by Myc- and Nod-LCOs after 6 h in our conditions, only 21 are activated in the *nsp1-1*, while 45 remain up-regulated in the *ram1-1* mutant (Additional file 2: Table S2). The strong NSP1-dependency of these Sym-LCO related genes indicates that many of them primarily respond to Nod-LCOs and are only cross-activated by Myc-LCOs, since their structure is similar. This is particularly relevant for the sMyc-LCOs, where an absence of RAM1 appears least important, while NSP1-dependency remains high (Fig. 4). Most Sym-LCO related genes thus seem to be activated by a route primarily controled by NSP1.

### Gene expression activation by Nod-LCOs displays a preferential requirement for the GRAS transcription factor NSP1

**Fig. 5:**
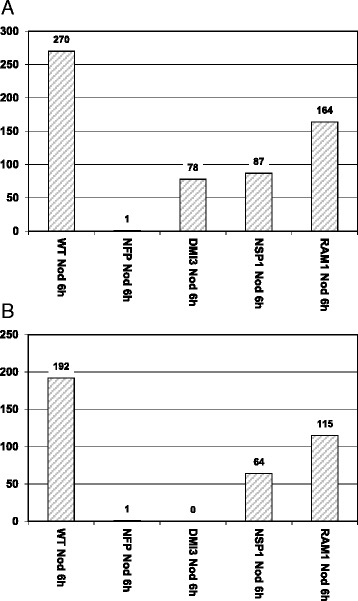
Nod-LCO related gene expression in the *M. truncatula* wild type and in four symbiotic mutants. **a** Number of genes at least 1.5-fold (*p* < 0.05) up-regulated in *M. truncatula* wild type A17 (WT), *nfp-1* (NFP), *dmi3* (DMI3), *nsp1-1* (NSP1)*,* and *ram1-1* (RAM1) plantlet roots challenged for 6 h with 10^−8^ M Nod-LCOs (Nod). These genes were not activated by Myc-LCOs at this cutoff (Additional file 3: Table S3). The number of genes induced by Nod-LCOs in the wild type still activated in four symbiotic mutants are shown to the right of the wild type column. **b** Genes still activated in *dmi3* mutants were subtracted (Additional file 4: Table S4) to highlight CSSP-dependent effects

Since our results provided evidence that genes co-activated by Myc- and Nod-LCOs show a higher requirement for NSP1 in comparison to RAM1, we assessed the NSP1- and RAM1-dependency of those 317 genes being activated at least 1.5-fold (*p* < 0.05) by Nod-LCOs, but not by Myc-LCOs after 6 h in *M. truncatula* A17 ([7], referred to as Nod-LCO related genes here, Additional file 3: Table S3). To achieve comparability with the results reported above, we used the same concentration (10^−8^ M) of Nod-LCOs as reported previously for the wild type [7], and also challenged plantlet roots for a duration of 6 h. To compare Nod-LCO activation of gene expression in the wild type to that in *nfp-1*, *dmi3*, *nsp1-1*, and *ram1-1* mutants, expression data had to be re-normalized across wild type and mutant conditions. This led to a slight change in the number of genes activated in the wild type at least 1.5-fold (*p* < 0.05) by Nod-, but not by Myc-LCOs (270 genes, Fig. 5).

Figure 5 shows that gene expression responses towards Nod-LCOs were reduced to similar levels as observed for Sym-LCO related genes in the *nfp-1*, *dmi3*, *nsp1-1,* and *ram1-1* mutants (appr. 99 %, 71 %, 68 %, 39 %, respectively), using the same cutoffs as mentioned above. As expected [37] and similar to Myc-LCO perception [7], these results revealed an almost complete dependency of the perception of Nod-LCO signals on NFP, and a predominant transduction of these nodulation-related signals via the CSSP. With respect to processes occuring downstream of DMI3, the requirement for NSP1 was much more pronounced than the requirement for RAM1 (Fig. 5). Similar to the situation observed for Sym-LCOs (Fig. 4), this effect was still evident after subtracting DMI3-independent genes and hence CSSP-independent responses (Additional file 4: Table S4, Fig. 5).

In conclusion, the RAM1-dependency of gene expression is comparably low not only for Sym-LCO, but also for Nod-LCO related transcription, indicating that Nod-LCO related genes are activated by a signaling pathway primarily controled by NSP1. However, a fraction of the Nod-LCO related genes identified in our conditions also depended on RAM1, a GRAS TF previously reported to be only required for Myc-signaling and mycorrhizal infection [42].

### RAM1 controls infection-related genes activated by nsMyc-LCOs during the pre-symbiotic stage

To address the question, if Myc-LCOs supplied at 10^-7/-8^ M primarily act as signals that pre-announce the presence of a mycorrhizal fungus, or if they are also able to activate genes connected to early fungal infection, we related our results to gene expression responses recorded from laser-microdissected AM tissues [30]. Although a comparison of microdissected cell types to the tissue samples studied here might include a bias, it allows us to obtain initial insights into overlapping expression patterns of genes responding to LCOs in the pre-symbiotic and the infection stage. In the laser-microdissection study used [30], gene expression in regions of mycorrhizal roots containing early infection stages as well as hyphopodia (APP) and in corresponding non-colonized areas (NAP) was analysed. Whereas in group A, 152 genes with a comparable expression in APP and NAP were identified that were also induced by Myc-LCOs, group B contained 126 genes up-regulated in APP [30]. Group A genes were thus thought to be root-expressed and activated by external Myc-signals before fungal contact, while group B genes were related to the initial intracellular infection [30]. It has to be noted that for both groups, a substantial portion of genes continued to be expressed in mature mycorrhizal roots, primarily in the arbuscule-containing cells [30].

Amongst the 152 genes from group A, 82 are activated not only by Myc-, but also by Nod-LCOs (Additional file 2: Table S2), indicating that these APP and NAP co-induced genes respond to LCOs of different microbial origin [30]. In line with the results for Sym-LCO related genes presented above, 43 of these are still up-regulated in response to Myc-LCOs independently of RAM1, while the NSP1-dependency remains higher (only 16 Myc-LCO induced genes, Additional file 2: Table S2).

Looking at the 126 genes up-regulated in regions of mycorrhizal roots containing early infection stages and hyphopodia [30], 11, 11, and 10 were already activated by sMyc-, nsMyc-, and s/nsMyc-LCOs in wild type plantlet roots, respectively (Additional file 2: Table S2). Due to redundancies, a total of 14 APP-induced genes were up-regulated by any Myc-LCO or Myc-LCO combination, providing evidence that a limited number of these infection-related genes responds to diffusible Myc-signals already at the pre-symbiotic stage. Interestingly, 25 APP-induced genes were activated by Nod-LCOs (Additional file 2: Table S2), including *MtVapyrin* [47, 48] and 10 of the 14 Myc-LCO induced genes mentioned above. This indicates a substantial cross-activation of infection-related genes by LCOs of different microbial origin, as reported by Camps et al. [34]. The co-activation by Nod-LCOs might also explain why nine of the 14 APP-induced genes up-regulated by Myc-LCOs are expressed independently of RAM1. Furthermore, the highest RAM1-dependency was found for the 11 APP-induced genes already activated by nsMyc-LCOs at the pre-symbiotic stage. Of these, only 4 were still up-regulated by nsMyc-LCOs in *ram1-1* mutants (Additional file 2: Table S2).

We now decided to focus on nsMyc-LCOs, since these lack the sulfate group that leads to a strong structural similarity between sMyc- and Nod-LCOs. Thus, gene expression responses to this most basic fungal LCO should be most instructive to identify genuine mycorrhization responses. To shed light on this, we had a look at all genes being activated by 10^−7^ M nsMyc-LCOs in the wild type, but not in *ram1-1* mutants. A total of 37 Myc-related (no activation by 10^−8^ M Nod-LCOs) and 50 Sym-related (co-activation by 10^−8^ M Nod-LCOs) genes fulfilled this criterion (Additional file 2: Table S2). Whereas the 37 Myc-related genes activated by nsMyc-LCOs displayed an almost absolute RAM1-dependency also towards sMyc-, s/nsMyc-, and Nod-LCOs (4 RAM1-independent genes found only for sMyc-LCOs, Additional file 2), the 50 Sym-related genes were completely RAM1-dependent only for their activation by nsMyc-LCOs. In contrast, a total of 28, 16, and 42 genes remained activated by sMyc, s/nsMyc-, and Nod-LCOs in *ram1-1* roots (Additional file 2: Table S2). On the one hand, this allows to conclude that the 37 Myc-related and 50 Sym-related genes up-regulated by nsMyc-LCOs are controled by a signaling pathway that strictly requires RAM1. On the other hand, in response to sMyc- or Nod-LCOs, a substantial fraction of these can be induced by an alternative pathway that that does not require RAM1 and is apparently not triggered by nsMyc-LCOs. This indicates that downstream of the CSSP, fine-tuning of gene expression activation travels along alternative roads, depending on the LCO variant initially perceived and possibly also on the epidermal cell type [10], where the CSSP is activated.

With respect to AM fungal infection, it is interesting to note that two of the 37 Myc-related and 14 of the 50 Sym-related genes activated by nsMyc-LCOs are up-regulated in regions containing early infection structures and hyphopodia (APP, [30]) or in arbuscule-containing cells (ARB, [30], Additional file 2: Table S2), while 12 and 22 were activated in whole AM roots, respectively ([28], Additional file 2: Table S2). Considering the RAM1-dependency of their activation by nsMyc-LCOs in the pre-symbiotic stage, it is thus possible that those genes are activated by nsMyc-LCO signals also during AM fungal contact and root infection.

## Discussion

Gene expression responses towards 10^-7/-8^ M of AM fungal and rhizobial LCOs in primary roots of the *M. truncatula* wild type as well as *nfp-1*, *dmi3*, *nsp1-1*, and *ram1-1* mutants demonstrated that during the pre-symbiotic stage, LCO-induced transcription strictly relies on the NFP receptor and largely on the calcium-calmodulin dependent kinase DMI3 (Figs. 2, 4, 5). This indicates that LCOs of whatever origin are perceived by the same LysM domain receptor kinase and that the majority of responses towards externally applied LCOs is controled by the CSSP. Due to the existence not only of a common receptor but also a common signal transduction pathway, specificity for Myc- or Nod-signals has to be generated downstream of it, where it is widely accepted that the GRAS transcription factors NSP1, NSP2, and RAM1 play an important role [4, 44]. These symbiotic regulators were shown to form NSP1/NSP2 and RAM1/NSP2 heterodimers [40, 42], respectively, and were thought to control either nodulation- (NSP1/NSP2) or mycorrhization-related (RAM1/NSP2) responses via parallel signaling pathways [42]. In line with this model, we have shown here that the activation of gene expression by Myc-LCOs during pre-symbiotic signaling within 6 h of application almost absolutely depends on RAM1, a GRAS transcription factor essential for root colonization and arbuscule formation [42]. Nevertheless, the NSP1 GRAS transcription factor was equally important for an up-regulation of gene expression by Myc-LCOs during the pre-symbiotic stage. It thus appears that the two symbiotic GRAS TFs are not only both essential for transcriptional reprogramming by Myc-LCOs at the lower concentrations used here, they also cannot complement each other. While the extent of NSP1-dependency was unexpected, our observation is supported by the re-assessment of NSP1 function by Delaux et al. [35] and Takeda et al. [36] as well as the strong NSP1-dependency observed for up to 100-fold higher Myc-LCO concentrations and shorter periods of application in an otherwise similar experimental setup [34]. Interestingly, from the 33 genes that Camps et al. [34] identified as co-activated by 10^-7/-8^ and 10^−6^ M Myc-LCOs, only 3, 14, 2, and 7 were still activated in the *nfp*, *dmi3*, *nsp1-1* and *ram1-1* background in our conditions, respectively (Additional file 2: Table S2), principally confirming a strict NFP-dependency, a predominant CSSP involvement, and a high RAM1 as well as NSP1-requirement for Myc-LCO induced transcription. With respect to NSP1, it might surprise that the strict dependency of Myc-LCO related gene expression on this GRAS TF contrasts with the comparably mild AM infection phenotype of *nsp1-1* mutants [35, 36]. This discrepancy can probably be explained by the fact that, reminiscent of the situation observed for *nfp* mutants, most pre-symbiotic responses to a low concentration of signal molecules mainly pre-announce, but not necessarily determine AM fungal contact and subsequent entry.

**Fig. 6:**
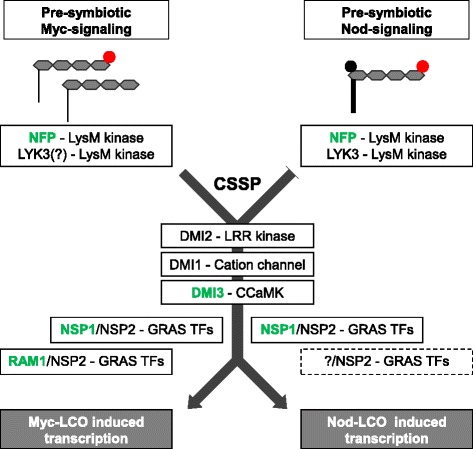
Model for the pre-symbiotic activation of host genes by microbial LCOs. This model integrates gene expression responses towards Nod- and Myc-LCOs in the wild type and in four symbiotic mutants at concentrations of 10^-7/-8^ M (Figs. 2, 4, 5). The two major pathways for a pre-symbiotic transduction of Nod- and Myc-LCO signals are shown. On top of the figure, LCO structures are depicted with four N-acetylamine residues. Sulfate groups of Nod- and sMyc-LCOs are shown in red, the acetate group typically present on Nod-LCOs in black, and differences in fatty acids by a different width. All components of signal perception and transduction studied in this work are shown in green. Other components of signal transduction reported in the literature are indicated in black. The partial dependency of Myc-LCO perception on LYK3 at high concentrations (10^−5^ M, [8]) is indicated by a question mark, since it is unknown if LYK3 is relevant at 10^-7/-8^ M as well. Whereas NSP1 and RAM1 are both essential for an up-regulation of Myc-LCO related genes not responding to Nod-LCOs, NSP1 is primarily required for the induction of genes by Nod-LCOs. The possible presence of additional GRAS TFs involved in activating Nod-LCO related gene expression is indicated by a dashed line. Abbreviations: *CSSP*, common symbiotic signaling pathway; *CCaMK*, Ca^2+^/calmodulin-dependent protein kinase; *LRR*, leucine-rich receptor; *TF*, transcription factor

During the pre-symbiotic stage, and despite the structural similarities between Myc- and Nod-LCOs that inevitably lead to a certain amount of cross-activiation of gene expression [34], in particular if sufficently high concentrations of less active LCO versions were used, host roots display characteristic gene expression patterns in response to microbial LCO signals ([7, 10, 34], Figs. 2, 4, 5). Our comparative analysis of gene expression responses during pre-symbiotic signaling presented here indicates that those Myc-LCO induced genes being either specifically or co-activated by Nod-LCOs still require NSP1, but are much less RAM1-dependent. It appears to us that the more Nod-LCO (co)-induced a gene is, the more NSP1- and the less RAM1-dependent it is. Obviously, Nod-LCO signals are thus primarily transduced via a signaling pathway controled by NSP1, which is in line with the initial nodulation-related function proposed for this TF [36–38]. However, a substantial portion of the Nod-LCO related genes identified here depends on the mycorrhiza-related [42] GRAS TF RAM1. It is possible that this fraction represents genuine Myc-LCO induced genes that were not identified as activated in our study, since the Myc-LCO concentrations used were too low or since alternative Myc-LCO molecules exist. In this case, these RAM1-dependent genes might have been cross-activated by the more active Nod-LCOs. On the other hand, it cannot be excluded that RAM1 plays a so far unknown role in Nod-LCO signaling and that, similar to the minor AM phenotype of *nsp1-1* mutants [35], *ram1-1* mutants exhibit a yet undetected, mild nodulation-related phenotype. While our results indicate that in the pre-symbiotic stage, signal transduction primarily requires different sets of GRAS TFs, it appears that a model where NSP1 solely controls the nodulation- and RAM1 the mycorrhization-related branch downstream of the CSSP is too simple. This is at least true for the pre-symbiotic responses to LCOs supplied for 6 h at 10^-7/-8^ M that we studied here. As shown by other reports [18, 45], there might well be additional signals that complete the picture of fungal recognition. In Fig. 6, a model for the pre-symbiotic activation of host genes by 10^-7/-8^ M microbial LCOs is proposed, focussing on the CSSP-dependent responses identified here. This model rests on the observation that downstream of the CSSP, NSP1 and RAM1 are both essential for the up-regulation of Myc-LCO related genes being not concomitantly activated by Nod-LCOs (Fig. 2), while NSP1 is primarily required for the induction of genes by Nod-LCOs (Figs. 4 and 5). From our results it is plausible that during the pre-symbiotic stage, transcriptional activation by Nod-LCOs predominantly requires NSP1, but at the same time, yet unidentified regulators, e.g. GRAS TFs, might still come into play. It also has be kept in mind that a substantial fraction of LCO-induced gene expression changes occurs independent of DMI3 (Figs. 2, 4, 5), suggesting the existence of parallel pre-symbiotic signaling pathways [34].

Apart from their involvement in Myc- and Nod-signaling, LCOs have a more basic function: the stimulation of root branching (RBS), thus leading to the formation of lateral roots [4]. It is likely that an initial mycorrhization-related, and subsequently a rather sophisticated nodulation-related signaling evolved from this basic developmental response. Whereas RAM1 was not essential for RBS mediated by Nod-LCOs, NSP1 was shown to be required both for RBS triggered by Nod- and sMyc-LCOs [4, 42]. This indicates that sulfated LCOs activate RBS primarily via NSP1, which is in line with our observation of a primarily NSP1-dependent pre-symbiotic activation of gene expression by sMyc and Nod-LCOs. For nsMyc-LCOs though, the NSP1- and RAM1-codependency of transcriptional activation observed here is in contrast to the RBS phenotype described in other studies [4]. This process was RAM1-dependent [4, 42], but NSP1-independent [4], indicating that nsMyc-LCO induced gene expression patterns are not primarily related to RBS. This conclusion is further supported by overlaps between nsMyc-LCO activation of transcription and gene expression during fungal infection.

Despite overlaps in gene expression reported here and on a broader scale by Camps et al. [34], obtained using 1 d younger seedling roots, it remains an open question whether Myc-LCO activated genes play an essential role during the colonization of roots by AM fungi or if they are mainly necessary to facilitate it. While NFP on the one hand is required for Myc-LCO dependent activation of Ca^2+^ spiking [10] and for almost all pre-symbiotic transcriptional responses towards Myc-LCOs supplied at 10^-7/-8^ M [7], *nfp-1* mutants on the other hand are not impaired in mycorrhization [49], indicating that at low concentrations, LCO signals are probably not essential for AM fungal entry and the infection of root cortical cells. However, our transcriptional analysis of the *nsp1-1* and *ram1-1* mutants demonstrated that downstream of the CSSP, the GRAS TFs RAM1 and NSP1 are required for the activation of gene expression by externally applied Myc-LCOs (Fig. 6). As far as processes downstream of the CSSP are concerned, these findings thus correlate pre-symbiotic responses towards Myc-LCOs with root infection, a process impaired in *nsp1-1* [35] and largely blocked in *ram1-1* [42] mutants. In this context, the RAM1-dependency in particular of the nsMyc-LCO induced gene expression is intriguing. Lower levels of and delayed fungal colonization were observed in the *ram1-1* background both in *M. truncatula* and in petunia. Moreover, degenerated arbuscules developed in the more infection-permissive *M. truncatula ram1-2* and the petunia *ram1-1* mutants upon high inoculum pressure, indicating that the degree of RAM1 knockout is important [45, 46]. It remains to be demonstrated, if transcriptional responses towards Myc-LCOs are different in *M. truncatula ram1-1* and *ram1-2* mutants as well.

It is an attractive hypothesis that apart from enhancing the formation of lateral roots to increase the chance of infection, Myc-LCOs pre-announce the presence of a beneficial fungus at a distance via the activation of the symbiotic GRAS TFs RAM1 and NSP1, that way priming host roots for a subsequent colonization by AM fungi. In addition, an interplay of Myc-LCOs and COs in the activation of Ca^2+^ spiking and downstream signal transduction is likely and can further mediate a multi-step recognition process from LCO-triggered pre-announcement via an initial physical contact to the actual infection [10]. Although a priming of root tissues for infection would certainly be helpful, it is probably not essential for colonization [50], which would explain the NFP-independency of AM formation.

LYK3, the second component of the heterodimeric Nod-LCO receptor, has recently been identified to be involved in Myc-LCO perception [8]. Following pre-symbiotic signaling that is likely mediated by a lower concentration of diffusible signals present in the area surrounding plant roots, AM fungal entry might be controled by elevated concentrations of Myc-LCOs, e.g. by activating the LYK3 receptor after accumulating in the local area underneath hyphopodia. This scenario is supported by the results of Zhang et al. [8] who reported that at 10^−5^ M, fungal LCOs induced only a reduced Ca^2+^ spiking in roots lacking LYK3. Apart from LCOs, short chain COs that do not require NFP to induce Ca^2+^ spiking [45], secreted fungal effectors [18], and plant-derived cutin monomers [51] probably play a role.

## Conclusions

Our integrated view of the transcriptional responses of the *M. truncatula* wild type and of four symbiotic mutants towards AM fungal and rhizobial LCOs sheds light on the pre-symbiotic reprogramming of host tissues by diffusible microbial signals. Well before a physical contact between the symbiotic partners, this pre-announcement probably informs the host about the presence of an appropriate symbiont in close vicinity of the root and in additon might prepare host roots for a subsequent colonization. While the initial perception and transduction of diffusible microbial signals occurs via the well-characterized LysM receptor kinase NFP and the CSSP, the interplay of transcriptional regulators acting immediately downstream of this signaling cascade is not yet fully understood. With respect to Myc-LCOs, we could clearly show that both the NSP1 and the RAM1 GRAS TFs are essential for almost all transcriptional responses towards Myc-LCOs at the pre-symbiotic stage, while Nod-LCOs predominantly rely on NSP1. This differential requirement for GRAS transcriptional regulators acting downstream of Ca^2+^ spiking and the CSSP can serve as a starting point to further unravel connections of the key jigsaw pieces that mediate the transduction of symbiotic signals in *M. truncatula* roots. These signals might even act in a sequential manner, as suggested by Nadal and Paszkowski [52]. Future experiments have to address the functional interplay and coordination of LCO and CO signals, prior to an opening of the gate for symbiotic guests. In this context, a dissection of the relevance of both AM fungal and plant signals for mycorrhizal infection remains a major challenge.

## Methods

### Sterilization of *M. truncatula* seeds and treatment of plantlets with symbiotic LCOs

Seeds of *M. truncatula* Gaertn cv. Jemalong genotype A17 (wild type), an *MtNFP* mutant (*nfp-1*, [49]), an *MtDMI3* mutant identified in a genetic screen of fast neutron mutagenised lines (*dmi3*, [53]), an *MtNSP1* (*nsp1-1*, [38]), and an *MtRAM1* mutant (*ram1-1*, [43]) were surface-sterilized and vernalized as reported in [54]. Seedlings used for Myc-LCO as well as the corresponding control treatments were grown for 5 days using the regime described in [7] on plates with half-strength Hoagland’s solution [55], whereas Nod-LCO treated and the corresponding control plantlets were grown on slant agar plates with N-free nutrient solution (NH-mix), as described in [56]. Upon treatment with LCOs, the seedlings were in a stage of development where they had just initiated their first trifoliate.

The treatment of 5 days old *M. truncatula* plantlet roots with LCOs was carried out as reported by [7], based on a procedure developed by [4]. In short, plantlet roots were placed in a tube containing 5 mL of the following solutions: sMyc-LCO (half-strength Hoagland’s solution pH 6.5 containing 10^−8^ M sMyc-LCOs), nsMyc-LCO (half-strength Hoagland’s solution pH 6.5 containing 10^−7^ M nsMyc-LCOs), s/nsMyc-LCO (half-strength Hoagland’s solution pH 6.5 containing 10^−8^ M sMyc-LCOs and 10^−7^ M nsMyc-LCOs), Myc control (half-strength Hoagland’s solution pH 6.5), Nod-LCO (NH-mix pH 7.5 containing 10^−8^ M Nod-LCOs), and Nod control (NH-mix pH 7.5).

Myc-LCOs synthesized via *Escherichia coli* cell factories [4] contained appr. 90 % tetramers (LCO-IV) and 10 % pentamers (LCO-V). Both sMyc- and nsMyc-LCOs were a mixture of compounds N-acylated by palmitic acid (C16:0) and oleic acid (C18:1∆9Z) in a 1:1 ratio. In case of Nod-LCOs, the treatment solution contained the major *S. meliloti* Nod-factor NodSm-IV-Ac-S (C16:2∆2E∆9Z) and appr. 10 % of the corresponding pentamer with the same O- and N-substitutions. LCOs were dissolved in 50 % (v/v) acetonitrile to obtain 10^−3^ M stock solutions, and were diluted further as described above. Separate batches of control plantlets were treated with Nod- and Myc-control solutions containing appropriate amounts of acetonitrile. To reach maximal comparability of Myc- and Nod-LCO related gene expression, LCO treatments of wild type and mutant roots were performed in parallel.

### RNA isolation and genome-wide expression profiling

To obtain tissues for transcriptome profiling, three biological replicates with 10 plantlets each were used. After 6 h of incubation, using the conditions described above, plantlets were harvested for each treatment and control condition. One mm of the root tip was removed, and the remaining 2 to 2.5 cm of the distal root region were cut off and directly frozen in liquid nitrogen. Ten fragments of each replicate were pooled and ground using lysing matrix D tubes in a FastPrep (MP Biomedicals). Total RNA isolation and DNase I digestion was performed via RNeasy kits (Qiagen) according to the manufacturer’s instructions. RNA preparations were quality-checked by spectrophotometry using a NanoDrop ND-1000 (Peqlab) and by capillary electrophoresis using a Bioanalyzer (Agilent). *Medicago* GeneChip hybridizations were carried out as described previously [28].

### Evaluation of *Medicago* GeneChip hybridizations

Cel files from *Medicago* GeneChip hybridizations were analysed using the Robin software [57]. Normalization was performed via the Robust Multichip Average (RMA) algorithm. Intensity values calculated for each probe set were log2-transformed and averaged across all three biological replicates. Log2 differences between the conditions compared were evaluated as described previously [7]. Original annotations of probes from *Medicago* GeneChips were replaced by updated annotations and functional classifications generated via the SAMS software [58], including matches to the current release of the *M. truncatula* genome [59, 60]. Since *Medicago* GeneChips are based on transcript and genomic sequences, the number of probe sets exceeds the number of genes represented. Nevertheless, we refer to genes instead of probe sets for reasons of simplicity.

### Availability of supporting data

The data sets supporting the results of this article are available in the Gene Expression Omnibus repository, http://www.ncbi.nlm.nih.gov/geo/query/acc.cgi?acc=GSE67167.

## Additional files

Additional file 1: Table S1
**a, b, c.** Compilation of gene expression responses in *M. truncatula* wild type A17 (WT), *nfp-1* (NFP), *dmi3* (DMI3), *nsp1-1* (NSP1)*,* and *ram1-1* (RAM1) plantlet roots challenged with 10^−8^ M sMyc-LCOs (sMyc), 10^−7^ M nsMyc-LCOs (nsMyc), a mixture of 10^−8^ M sMyc- and 10^−7^ M nsMyc-LCOs (s/nsMyc), and 10^−8^ M Nod-LCOs (Nod). Treatment of *M. truncatula* wild type roots was performed for 6 or 24 h, respectively, whereas mutants were challenged for 6 h. Expression profiles of wild type roots towards Myc- and Nod-LCOs reported by [7] were included to facilitate comparisons to the global responses in the symbiotic mutants mentioned above. Log_2_ average expression values (Log AveExpr), log_2_ fold changes (logFC) for each LCO vs. control treatment, and associated p-values are indicated. Log_2_ fold changes are coloured as follows: red, log_2_ fold change larger than 1.0; orange, log_2_ fold change between 0.6 and 1.0; green, log_2_ fold change below −1.0. Updated annotations for all *Medicago* probes including correspondences to release 4.0 of the *M. truncatula* genome and names of known *M. truncatula* genes are given. The file was split in three parts for reasons of size. (ZIP 43742 kb) Additional file 2: Table S2. Gene expression responses in *M. truncatula* wild type A17 (WT), *nfp-1* (NFP), *dmi3* (DMI3), *nsp1-1* (NSP1)*,* and *ram1-1* (RAM1) plantlet roots challenged with 10^−8^ M sMyc-LCOs (sMyc), 10^−7^ M nsMyc-LCOs (nsMyc), a mixture of 10^−8^ M sMyc- and 10^−7^ M nsMyc-LCOs (s/nsMyc), and 10^−8^ M Nod-LCOs (Nod). Individual sheets contain subsets of genes, as defined in the captions. Log_2_ average expression values (Log AveExpr), log_2_ fold changes (logFC) for each LCO vs. control treatment, and associated p-values are indicated. Log_2_ fold changes are coloured as follows: red, log_2_ fold change larger than 1.0; orange, log_2_ fold change between 0.6 and 1.0; green, log_2_ fold change below -1.0. Updated annotations for all *Medicago* probes including correspondences to release 4.0 of the *M. truncatula* genome and names of known *M. truncatula* genes are given. (XLSX 1289 kb) Additional file 3: Table S3. Gene expression responses in *M. truncatula* wild type A17 (WT), *nfp-1* (NFP), *dmi3* (DMI3), *nsp1-1* (NSP1)*,* and *ram1-1* (RAM1) plantlet roots challenged with 10^−8^ M sMyc-LCOs (sMyc), 10^−7^ M nsMyc-LCOs (nsMyc), a mixture of 10^−8^ M sMyc- and 10^−7^ M nsMyc-LCOs (s/nsMyc), and 10^−8^ M Nod-LCOs (Nod). Individual sheets contain subsets of genes, as defined in the captions. Log_2_ average expression values (Log AveExpr), log_2_ fold changes (logFC) for each LCO vs. control treatment, and associated p-values are indicated. Log_2_ fold changes are coloured as follows: red, log_2_ fold change larger than 1.0; orange, log_2_ fold change between 0.6 and 1.0; green, log_2_ fold change below −1.0. Updated annotations for all *Medicago* probes including correspondences to release 4.0 of the *M. truncatula* genome and names of known *M. truncatula* genes are given. (XLSX 2932 kb) Additional file 4: Table S4. Ten subsets from Additional file 3: Table S3, where genes activated at least 1.5-fold (*p* < 0.05) in *dmi3* (DMI3) mutants were subtracted, as defined in the captions. Log_2_ fold changes (logFC) are coloured as follows: red, log_2_ fold change larger than 1.0; orange, log_2_ fold change between 0.6 and 1.0; green, log_2_ fold change below −1.0. Updated annotations for all *Medicago* probes including correspondences to release 4.0 of the *M. truncatula* genome and names of known *M. truncatula* genes are given. (XLSX 2260 kb)
